# Porcine granulosa cell transcriptomic analyses reveal the differential regulation of lncRNAs and mRNAs in response to all-trans retinoic acid *in vitro*

**DOI:** 10.5713/ab.24.0363

**Published:** 2024-08-26

**Authors:** Jinzhu Meng, Xiuwen Chen, Huabiao Wang, Yixuan Mi, Runsheng Zhou, Hongliang Zhang

**Affiliations:** 1 College of Veterinary Medicine, Hunan Agricultural University, Changsha 410128, China; 2 Guizhou Provincial Key Laboratory for Biodiversity Conservation and Utilization in the Fanjing Mountain Region, Tongren University, Tongren 410128, China

**Keywords:** All-trans Retinoic Acid (ATRA), Follicular Development, Granulosa Cells, Pig

## Abstract

**Objective:**

The active metabolite of vitamin A, all-trans retinoic acid (ATRA), is involved in the proliferation and differentiation of granulosa cells, and promotes the follicular development, oocyte maturation, and ovulation in mammals. This study aims to investigate the ATRA induced potential long noncoding RNAs (lncRNAs) that regulate the expression of genes associated with granulosa cell proliferation and follicular development.

**Methods:**

The lncRNA and mRNA profiles of porcine granulosa cells from ATRA treatment and control group *in vitro* were constructed through RNA sequencing. Meanwhile, the sequencing data were verified using quantitative polymerase chain reaction (qPCR).

**Results:**

A total of 86 differentially expressed lncRNAs and 128 differentially expressed genes (DEGs) were detected in granulosa cells after ATRA treatment. The quantitative real-time PCR (qRT-PCR) results were consistent with the RNA-seq data. Functional annotation analysis revealed that the DEGs were remarkably enriched in ovary function and reproduction which contained FoxO, Hippo, Oocyte meiosis, mammalian target of rapamycin signaling pathway, as well as several pathways associated with hormone regulation like oxytocin signaling pathway and steroid hormone biosynthesis. Moreover, an interaction network of lncRNAs and their cis-target DEGs was constructed, and 7 differentially expressed lncRNAs and 6 cis-target DEGs were enriched in ovarian steroidogenesis and reproduction.

**Conclusion:**

These findings expand the lncRNA catalogue and provide a basis for further studies on the mechanism of ATRA-mediated lncRNA regulation of follicular development in pigs.

## INTRODUCTION

In female mammals, follicular ovulation or atresia is a normal physiological phenomenon. During an estrus cycle, despite a cluster of antral follicles induced to recruit, more than 99% of the follicles in ovary will undergo atresia at different stages of follicle development [1,2]. This phenomenon is prevalent in the ovaries of cattle [3], goat [4], pig [5], and other mammals [6]. The apoptosis of granulosa cells reportedly is the key factor leading to follicular atresia [7]. Previous study has shown that the hormones and factors (follicle stimulating hormone [FSH], luteinizing hormone [LH], and insulin-like growth factor [IGF]) promoted estradiol secretion by granulosa cells, offering a stable internal environment for oocyte development and maturation [8,9]. Therefore, a shift in the apoptotic balance of granulosa cells may alter the fate of follicular atresia caused by granulosa cell apoptosis.

All-trans retinoic acid (ATRA), a natural component and representative physiologically active metabolite of vitamin A, has been implicated in a variety of physiological processes in reproduction, including regulating the meiosis initiation of germ cells [10], enhancing oocyte maturation *in vitro* [11], and accelerating the differentiation of follicles [12]. It has been reported that ATRA enriched in granulosa cumulus cells promoted oocyte maturation by reducing apoptosis of granulosa cumulus cells in goat ovaries [13]. Moreover, ATRA possess a certain regulatory effect on ovarian development related pathways. *In vitro*, ATRA enhances the follicle and granulosa cell competence by altering the expression of FSH receptor (FSHR) and LH receptor (LHR) [14]. Studies in sows found that vitamin A addition increased the litter size and the embryonic development ability before ovulation [15]. These findings showed that vitamin A and its derivative ATRA might exert a crucial role on follicle development and oocyte maturation.

Long non-coding RNA (lncRNA) is a kind of RNA which has no protein-coding potential [16]. Nevertheless, LncRNA has the ability to regulate gene expression in epigenetics, transcriptional, and post-translational levels, thereby affecting many biological processes, including cell proliferation, differentiation, and apoptosis [2]. Studies in mouse embryonic palate mesenchymal cells found that ATRA treatment promoted the *lncRNA-Meg3* upregulation, which interacts with the Smad2 protein to inhibit Smad-dependent pathway activation and cell proliferation [17]. In addition, *lncRNA-H19* mediated ATRA-induced cleft palate by down-regulating the expression of IGF2 in mice [18]. Yet, a systematic analysis of ATRA-mediated lncRNAs expression in granulosa cells associated with follicular development, especially in pig, has not been reported. In this study, RNA-seq was performed on porcine granulosa cells to identify potential differentially expressed lncRNAs and mRNAs associated with ATRA-mediated follicular development. Our findings will provide new insights for exploring the molecular mechanism of ATRA-mediated lncRNA regulation of follicular development in pigs.

## MATERIALS AND METHODS

All animal experimental procedures were performed in accordance to the ethical recommendations of Hunan Agricultural University, China (Approval ID: 432035233). This study does not contain any studies with human subjects or with rodent vertebrates and informed consent is not applicable.

### Granulosa cells recruits and culture *in vitro*

Ovaries were obtained from 20 female Large White pigs at Changsha Red Star Shengye slaughterhouse (Changsha, Hunan, China). Follicles (3 to 6 mm in diameter) were dissected free from ovarian stroma and washed in physiological saline containing 1% penicillin-streptomycin for a few seconds. Follicular fluid was aspirated into 15 mL conical centrifuge tubes (Corning, New York USA), granulosa cells were isolated by centrifugation at 1,100×g for 7 min and resuspended in red blood cell lysis buffer for 2 min followed by centrifugation at 1,100×g for 7 min. After discarding the supernatant, the cell pellets were resuspended in sterile phosphate-buffered saline containing 1% penicillin-streptomycin followed by centrifugation at 1,100×g for 7 min. Finally, the granulosa cells were resuspended with complete medium (DMEM/F-12; Gibco, 11330032; Thermo fisher scientific, Waltham, MA, USA) containing 10% fetal bovine serum and 1% penicillin-streptomycin and were incubated under a humidified atmosphere at 37°C with 5% CO_2_. The ATRA (Sigma, St. Louis, MO, USA) was employed at a concentration of 1 nM for granulosa cell treatment *in vitro*. Dimethyl sulfoxide (DMSO) (Sigma, USA) was used as a solvent for ATRA, and the control group was treated with DMSO (0.01% v/v) without ATRA. The treatments were maintained for 24 h in the experimental setup.

### RNA extraction and RNA-sequencing

Total RNA extraction from granulosa cells (n = 4, single cell samples containing 5×10^6^ cells) of control and ATRA treatment group were utilized TRIzol (Invitrogen, Carlsbad, CA, USA). RNA quality and quantity were assessed using 1% agarose gels and NanoPhotometer spectrophotometer (IMPLEN, Munich, Bavaria, Germany), respectively. Individual sequencing libraries employed 3 μg RNA per sample, were conducted on an Illumina Hiseq TM 4000 platform (Illumina, San Diego, CA, USA) and the raw reads were generated.

### Transcriptome data assembly

The raw reads were filtered into clean reads through removing adaptor contamination, low quality bases and undetermined bases using Cutadapt (v1.10). Further, FastQC (http://www.bioinformatics.babraham.ac.uk/projects/fastqc/) was used to verify the sequence quality of the clean reads, which were mapped to the reference genome (*sus scrofa* v96) by Bowtie2 (v2.2.9) and Hisat2 (v2.0.4). Next, StringTie (v1.3.0) was used to assemble the reads that mapped to reference genome of each sample. Then, a comprehensive transcriptome was reconstructed by merging every granulosa cell transcriptome using perl scripts (v5.10.0). Finally, the expression levels of all transcripts were estimated via StringTie (v1.3.0) and edgeR.

### Identification of lncRNAs

Transcripts that overlapped with known mRNAs, transcripts shorter than 200 bp, and exons greater than two were discarded. Next, coding potential calculator (CPC, v0.9-r2) and coding-non-coding index (CNCI, v2.0) were utilized to predict transcripts with coding potential, and the transcripts with CPC score <−1 and CNCI score <0 were removed. The remaining transcripts were considered as lncRNAs.

### Identification of differentially expressed lncRNAs and mRNAs

Transcripts or gene expression profiles were generated based on the Fragments per kilobase of exon model per million mapped reads (FPKM) utilizing Cuffdiff (v2.2.1). The DESeq2 of R package (3.12.1) was performed to select the differentially expressed lncRNAs and mRNAs using a cut-off |log2(fold change)|≥1 and adjusted p<0.05. The expression-based sample clustering and principal component analysis were performed using DESeq2 R package (1.10.1).

### Target gene prediction and functional enrichment analysis

LncRNAs may play a cis role acting on neighboring target genes, so we selected the coding genes in 100,000 bp upstream and downstream of each differentially expressed lncRNA as cis-target genes by python script. Then, gene ontology (GO) annotations, Kyoto encyclopedia of genes and genomes (KEGG) enrichment of the target genes for lncRNAs and differentially expressed mRNAs were conducted via the cluster Profiler R package, and adjusted p<0.05 was the significance threshold (enrichment). To further investigate the interactions between the lncRNAs and differentially expressed mRNAs, we selected the target genes that overlapped with differentially expressed mRNAs and their source lncRNAs to construct PPI network. The network related to granulosa cell proliferation and female reproduction was sorted out based on their enriched GO and KEGG terms with key words, and visualized using 3.7.1 (https://cytoscape.org/, USA).

### Quantitative analysis of lncRNA and mRNA expression

Following total RNA (1 μg) extraction from ovaries and granulosa cells, we generated cDNA using EasyScript All-in-One First Strand cDNA Synthesis SuperMix for qPCR (One-Step gDNA Removal) (AE341-02; Transgen, Beijing, China), and performed quantitative polymerase chain reaction (qPCR) via a 20 μL reaction volume with 10 μL 2×Transstart Tip Green qPCR super mix (AQ141-02; Transgen, China), 0.8 μL forward and reverse primers, 4 μL cDNA, and 5.2 μL RNase and DNase-free water, using a LightCycler 480 platform (Roche, Basel, Switzerland) adjusted to 94°C for 30 s, 40 cycles of 94°C for 10 s, 60°C for 30 s, and 72°C for 10 s. Glyceraldehyde-3-phosphate dehydrogenase served as an endogenous control, and relative gene expression was computed with the 2^−ΔΔCT^ formula. Significance was determined using Student’s t-test, SPSS 22.0. The employed primer sequences are detailed in Table 1.

### Statistical analysis

Data were assessed via SPSS Statistics 22.0 software (SPSS Software, Inc., Chicago, IL, USA), and are provided as means ±standard error of the mean. Data comparison employed one-way analysis of variance test, with subsequent Dunnett’s multiple comparison test and significant difference was inferred for p<0.05 and extremely significant difference p<0.01.

## RESULTS

### Quality analysis of RNA sequencing

As shown in Table 2, a total of 733,403,634 raw reads were generated from the eight cell samples, and 668,220,004 clean reads were obtained after filtering. The Q20 and Q30 values of each sample were greater than 99.93% and 97.49%, respectively. The average ratio of clean reads and unique reads mapped to the reference genome (*sus scrofa* v96) were reached 80.91% and 70.43% respectively.

### Identification of lncRNAs and mRNAs in porcine ovarian granulosa cells

**Figure 1:**
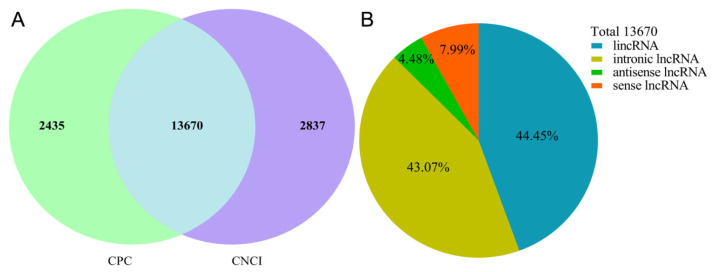
Identification of the lncRNAs in porcine granulosa cells after ATRA treatment. (A) The lncRNAs identified from the intersection of CPC and CNCI. (B) Classification of the 13,670 lncRNAs identified. ATRA, all-trans retinoic acid; CPC, coding potential calculator; CNCI, coding-non-coding index.

After removing the known mRNA and transcripts less than 200 bp, the remaining transcripts were utilized to screen lncRNAs by CPC and CNCI software (Figure 1A). In total, 13,670 lncRNAs (402 known lncRNAs and 13,268 novel lncRNAs) were identified, containing 6,076 lincRNAs (44.45%), 5,888 intronic lncRNAs (43.07%), 1,092 sense lncRNAs (7.99%), and 614 antisense lncRNAs (4.48%) (Figure 1B). In addition, we also obtained 36,797 annotated mRNAs for subsequent analysis.

**Figure 2:**
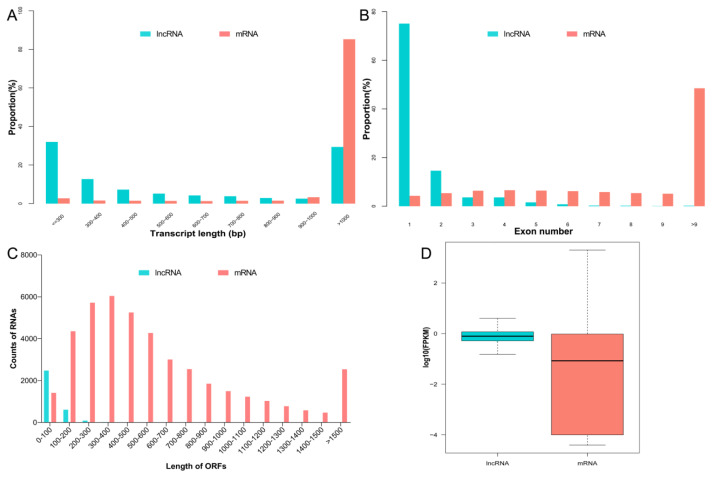
Identification of lncRNAs and mRNAs in porcine granulosa cells after ATRA treatment. (A) Length distribution of the lncRNAs and mRNA transcripts. (B) Exon number distribution of the lncRNAs and mRNA transcripts. (C) ORF length distribution of the lncRNAs and mRNA transcripts. (D) FPKM levels of the lncRNAs and mRNAs in porcine ovarian granulosa cells after ATRA treatment. ATRA, all-trans retinoic acid; ORF, open reading frames; FPKM, fragments per kilobase of exon model per million mapped reads.

The lncRNA length mainly distributed below 500 bp, while the mRNA transcript length mostly distributed over 1,000 bp, and the ratio of lncRNAs and mRNA transcripts declined due to the increasing length (Figure 2A). Furthermore, the lncRNAs mainly contained one to two exons, whereas the exon numbers of mRNA transcripts were mostly greater than 9 (Figure 2B). In addition, the average length of open reading frames (ORF) of the lncRNA transcripts (about 81 bp, on average) was shorter than that of the mRNA transcripts (about 442 bp, on average) (Figure 2C). Finally, we found the average expression levels of lncRNAs were lower than that of mRNAs (Figure 2D).

### Differential expression pattern of lncRNAs and mRNAs

**Figure 3:**
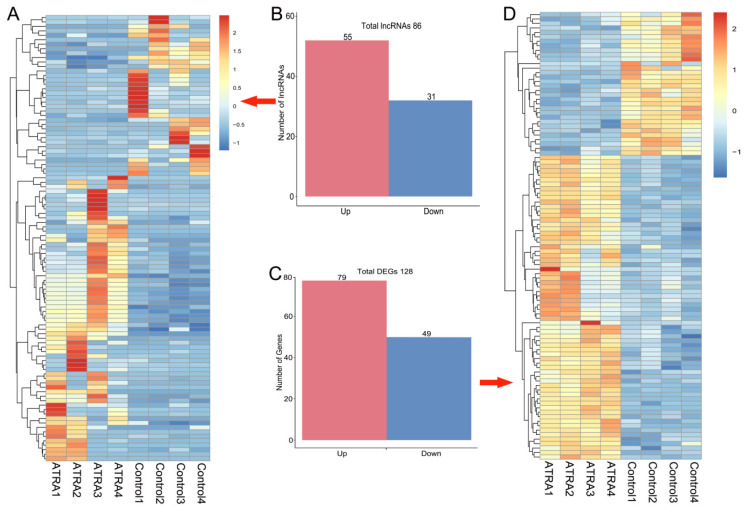
Differentially expressed analysis of lncRNAs and DEGs in porcine granulosa cells after ATRA treatment. (A) The heatmap of the significantly expressed lncRNAs. (B) Number of the differentially expressed lncRNAs. (C) Number of the DEGs. (D) The heatmap of the significantly expressed mRNAs. DEGs, differentially expressed genes; ATRA, all-trans retinoic acid.

In all, 86 differentially expressed lncRNA transcripts (55 up regulated and 31 down regulated) (Figure 3B; Supplementary Table S1) and 128 differentially expressed mRNA transcripts (79 up regulated and 49 down regulated) (Figure 3C; Supplementary Table S2) were identified after ATRA treatment following the criteria (corrected p-value <0.05, FPKM >0.5, and |log2(fold change)|≥1). The expression patterns of differentially expressed lncRNAs (Figure 3A) and DEGs (Figure 3D) were exhibited via systematic clustering analysis in the samples.

### Gene ontology and Kyoto encyclopedia of genes and genomes analysis of differentially expressed genes

**Figure 4:**
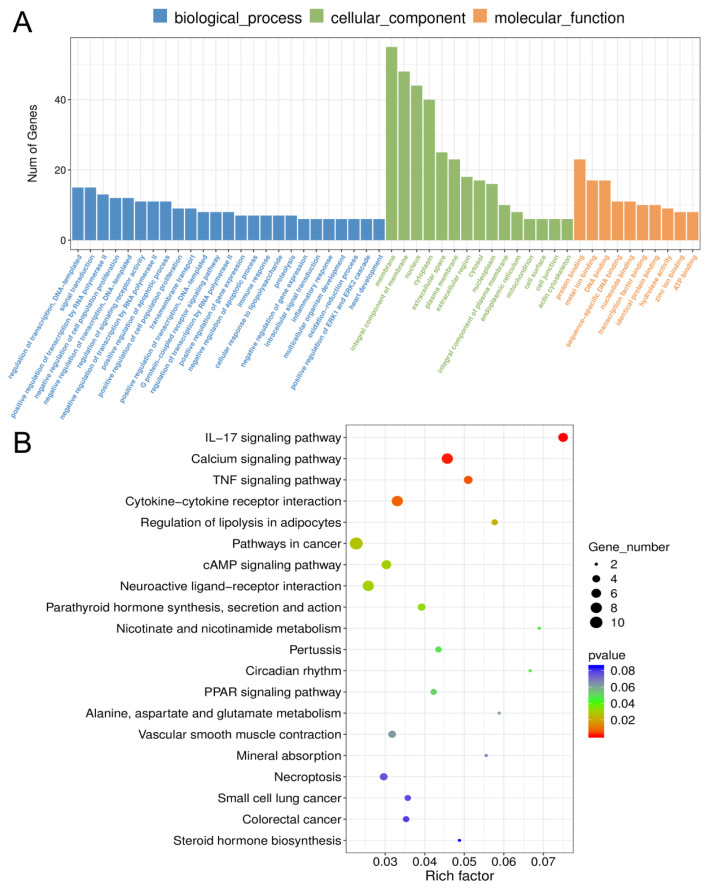
GO and KEGG enriched terms of the differentially expressed genes in porcine granulosa cells after ATRA treatment. (A) Statistics of the enriched GO terms for differentially expressed genes. (B) Statistics of the top 20 KEGG pathways for differentially expressed genes. The rich factor was calculated using the number of enriched differentially expressed mRNAs divided by the total number of background genes in the corresponding pathway. GO, gene ontology; KEGG, Kyoto encyclopedia of genes and genomes; ATRA, all-trans retinoic acid. A pathway with a corrected p-value <0.05 is significantly overrepresented.

GO and KEGG analysis were performed on 128 DEGs. For GO analysis, the 128 DEGs were markedly enriched in 50 GO terms (Figure 4A) which can be divided into biological process, cellular component, and molecular function. In the category of biological process, DEGs were most enriched in regulation of transcription (GO:0006355), signal transduction (GO:0007165), and positive regulation of transcription by RNA polymerase II (GO:0045944) and so forth. In cellular component category, membrane (GO:0016020), integral component of membrane (GO:0016021), nucleus (GO:000 5634) were most significantly enriched. In addition, DEGs enriched in protein binding (GO:0005515), DNA binding (GO:0003677), metal ion binding (GO:0046872) were the most dominant which categorized at molecular function.

Furthermore, 155 pathways were annotated based on KEGG enrichment analysis (Supplementary Table S3). Among them, pathways related to ovary function and reproduction which contained FoxO (ko04068), Hippo (ko04390), Oocyte meiosis (ko04114), mammalian target of rapamycin signaling pathway (ko04150), as well as several pathways associated with hormone regulation like Oxytocin signaling pathway (ko04921) and Steroid hormone biosynthesis (ko00140). The top 20 significantly enriched pathways are shown in Figure 4B, which revealed that differentially expressed mRNAs were mainly involved in interleukin-17, calcium, and tumor necrosis factor signaling pathway.

### Screening of potential functional lncRNAs related to porcine reproduction

**Figure 5:**
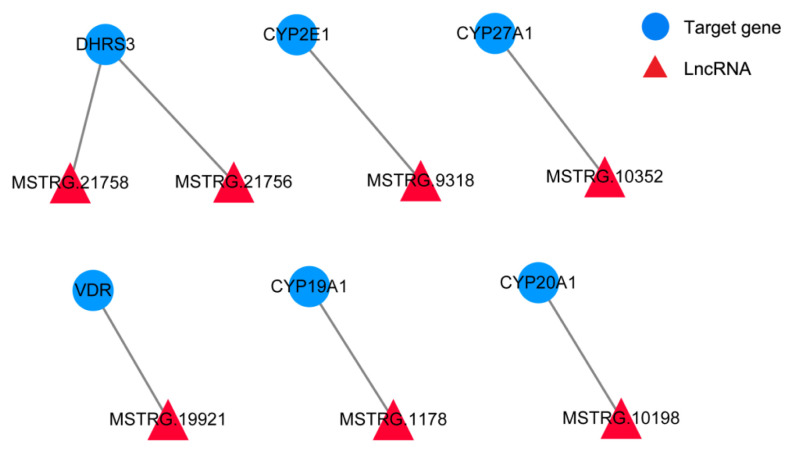
Potentially functional lncRNAs and their predicted targeted differentially expressed genes compose this interactive network.

We constructed the interaction network of lncRNAs and their cis-target genes, and found 14 DEGs were cis-targeted with 15 differentially expressed lncRNAs, which suggested that these 14 genes might be influenced by the lncRNAs via their cis-regulatory activities. To explore the lncRNAs related to cell proliferation, hormone secretion, and reproduction, the 15 genes were mapped into the GO and KEGG pathway database, respectively. The cis-target genes are enriched in GO terms containing female gonad development, steroid hormone receptor activity, vitamin D binding, uterus development, and regulation of retinoic acid receptor signaling pathway and KEGG pathways including steroid hormone biosynthesis and ovarian steroidogenesis (Supplementary Table S4). Based on the GO and KEGG pathway analysis results, seven differentially expressed lncRNAs and 6 cis-target DEGs were enriched in ovarian steroidogenesis and reproduction and are shown in Figure 5. The network provides candidate lncRNAs related to porcine reproduction. Interestingly, most of the interacting genes were upregulated in granulosa cells after treated with ATRA.

### Verification of differentially expressed lncRNAs and mRNAs

**Figure 6:**
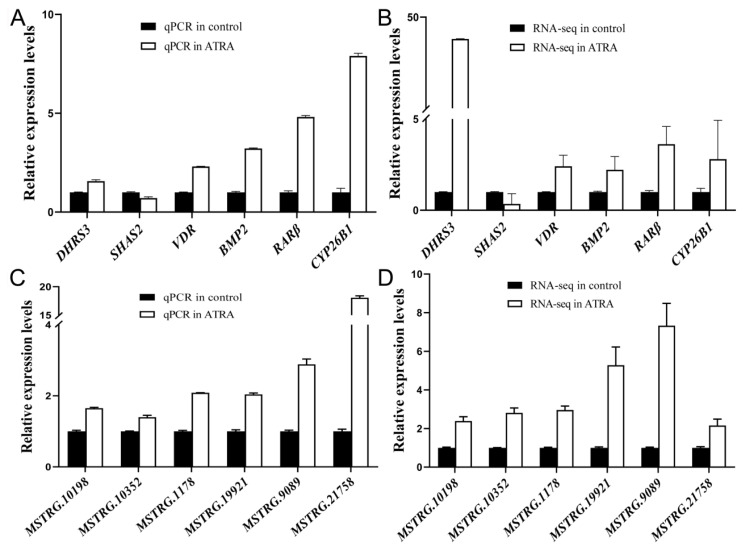
Verification of the expression of differentially expressed lncRNAs and genes in porcine granulosa cells after ATRA treatment. (A, B) The relative expression level of 6 differentially expressed genes in different groups determined by qPCR and RNA-Seq, respectively. (C, D) The relative expression level of 6 differentially expressed lncRNAs in different groups determined by qRT-PCR and RNA-Seq, respectively. The qPCR data were represented as the mean±standard error of the mean (n = 4). The RNA-seq data were represented as the FPKMs of each transcript (n = 4). ATRA, all-trans retinoic acid; qPCR, quantitative polymerase chain reaction; FPKMs, fragments per kilobase of exon model per million mapped reads.

Six differentially expressed lncRNAs and DEGs related to cell proliferation, hormone secretion, and reproduction were selected and their expression levels in the two groups were verified through qPCR, respectively. The results confirmed by the expression levels of the six lncRNAs and DEGs were consistent with the RNA-seq data (Figure 6). Therefore, these data suggest that the RNA-seq results are reliable and can represent the virtual expression pattern in porcine ovarian granulosa cells after treated with ATRA.

## DISCUSSION

Granulosa cumulus cells are the primary sites of retinoid uptake and ATRA biosynthesis, lncRNAs and genes which regulated by ATRA may promote follicle development, oocyte maturation and are essential in fertilization [19]. Here, we conducted genome-wide analyses to identify mRNAs and lncRNAs that were differentially expressed in porcine ovarian granulosa cells associated with ATRA-mediated follicular development. In total, 13,670 lncRNAs and 36,797 annotated mRNAs were identified. Most of the lncRNAs were reportedly located adjacent to the protein-coding genes [20], which suggest that these lncRNAs have synergistic relationships with mRNAs. Furthermore, the lncRNA length mainly distributed below 500 bp, contained one to two exons, and the average length of ORF was 81 bp which shorter than that of the mRNA transcripts, indicating that the data confidence was reliable and potential specific lncRNAs and mRNAs were identified after ATRA treatment.

In the current study, we detected 86 differentially expressed lncRNA transcripts and 128 differentially expressed mRNA transcripts after ATRA treatments, indicating these RNAs are directly or inversely associated to ATRA sensitivity. Several studies have already discovered differential gene expression in cancer cell-lines based on global gene expression analysis, such as *CYP26B1*, *DHRS3*, and *TINAGL1* were up-regulated by ATRA [21]. Of particular interest was that the expression of *CYP26B1*, *DHRS3*, and *TINAGL1* was enhanced in granulosa cells treated with ATRA, which have not been detected in the context of ATRA-sensitive granulosa cells before. The up-regulation of *CYP26B1* and *DHRS3*, indicating that ATRA causes marked impact on the metabolism of endogenous vitamin-A in granulosa cells. In fact, ATRA oxidation was catalyzed into an inactive oxo-derivative through a protein encoded by *CYP26B1* [22], whereas, retinaldehyde was reduced into retinol through NADPH-dependent enzyme encoded by *DHRS3* [23]. From a functional point of view, *TINAGL1*, the corresponding protein regulates EGFR signaling pathway [24], and suppress the spontaneous onset of apoptosis and induce proliferation of granulosa cells, follicular development and ovulation thereby [25].

Based on the GO enrichment analysis, 128 DEGs in granulosa cells were markedly enriched in regulation of transcription, membrane, protein binding and so forth. It is well known that the expression of vitamin D receptor (VDR) and vitamin D-metabolizing enzyme CYP27A1 has been widely demonstrated in ovaries [26]. VDR heterodimerizes with retinoic acid X receptor and acts as a transcription factor for genes with vitamin D response elements after translocation to the nucleus, and its expression in injured cells is often decreased [27]. The expression of *CYP27A1* mRNA that encodes an enzyme involved in cholesterol catabolism to oxysterols, thereby modulates sterol biosynthesis [28]. Interestingly, *VDR* and *CYP27A1* were abundantly enriched in most of the GO terms of current study, which suggested that these genes might involve in the ATRA-mediated granulosa cell proliferation and follicular development.

The KEGG analysis revealed that CYP19A1 and CYP2E1 take part in the steroid hormone biosynthesis and ovarian steroidogenesis pathways that might play an essential role in ATRA-induced follicular development. Steroid hormones play a key role in multiple follicle cells in the entire growth phase. Notably, *CYP19A1* is a marker gene related to steroids, which has the function of dynamically indicating the secretion of steroid hormones [29]. In addition, FSH interacts with its homologous receptor to induce *CYP19A1* expression, converting androgens from theca cells into estradiol, which drives follicle development and leads to ovulation [30]. *CYP2E1*, encodes a detoxifying enzyme was found to be significantly up-regulated in developing oocytes, and was considered to be related to DNA replication and steroid biosynthesis as well as those involved with oxidative stress-related injuries.

It has been reported that lncRNAs may be closely involved in the regulation of follicle growth and maturation along with mRNAs. For instance, the promoter activity was enhanced by *lncRNA*-*AMH2* to regulate the expression of *AMH2* in ovarian granulosa cells of mouse [31]. Moreover, in polycystic ovarian syndrome of rat, *lncRNA-HOTAIR* increases the expression of *IGF1*, which ultimately aggravates endocrinopathy and granulosa cell apoptosis [32]. Therefore, we constructed the lncRNA target gene interaction networks by integrating differentially expressed lncRNAs, DEGs and their co-regulatory relationships. The upregulated gene *DHRS3* was actioned by its targeted lncRNAs (*MSTRG.21756* and *MSTRG.21758*), and the upregulated *lncRNAs MSTRG.9318*, *MSTRG.10352*, *MSTRG.19921*, *MSTRG.1178*, and *MSTRG.10198* target *CYP2E1*, *CYP27A1*, *VDR*, *CYP19A1*, and *CYP20A1*, respectively. In addition, the lncRNA target DEGs (*DHRS3*, *CYP2E1*, *CYP27A1*, *VDR*, *CYP19A1*, and *CYP20A1*) are currently considered signaling molecules with a proven role in regulating female reproduction [26,29,33,34], which indicated that differentially expressed lncRNAs and their target DEGs might play a crucial role in the biofunction of ATRA-mediated granulosa cell proliferation and follicular development.

In conclusion, 86 differentially expressed lncRNAs and 128 DEGs were identified in porcine ovarian granulosa cells after ATRA treatment *in vitro*. Based on GO and KEGG database, the target DEGs of differentially expressed lncRNAs were annotated at many biological processes and pathways related to granulosa cell proliferation and follicular development. The upregulated lncRNAs *MSTRG.21756*, *MSTRG. 21758*, *MSTRG.9318*, *MSTRG.10352*, *MSTRG.19921*, and *MSTRG.1178* might release potential abilities by regulating their target genes that involved in regulating female reproduction. Our findings will provide new insights for exploring the molecular mechanism of ATRA-mediated lncRNA regulation of follicular development in pigs.

## Figures and Tables

**Table 1:** Primer sequences for quantitative polymerase chain reaction

Gene name/lncRNA	Primer sequence (5′-3′)	Product length (bp)
*MSTRG.10198*	F: CCAAGACAGTGAATGGGGCT R: GACTGCATGAAGAGTCGGCA	235
*MSTRG.10352*	F: CTGCACTTACACCAGCTCCA R: CGTACTGGGTACTTGCCCTC	146
*MSTRG.21758*	F: GTGCAGCTCAATCAGACCCT R: TCCCTGCGTCCTGCTTTTAG	166
*MSTRG.1178*	F: GCTACCCGGTGAAAAAGGGA R: GCGTCTCAGAAGTGTGACCA	218
*MSTRG.19921*	F: CCTCCGTTATGAGGAGTTGCT R: GCTATTTCTCCTGCCTGCCT	144
*MSTRG.9089*	F: AATGAGAAGGAGTGGCACCG R: CCGAAGGGCAAGTAGCTCAA	104
*VDR*	F: CAGCCAGCACTTCCTTACCT R: CGTCCGTCAGGATGAACTCC	279
*SHAS2*	F: TTTGGAGCACCGGAAAATGAAA R: TGAATTGTCCCTGCCCATGA	235
*DHRS3*	F: GAGGAGATTCGGCAGATGGG R: CACGGAGTTGAGACACACGA	288
*CYP26B1*	F: GAGCTACCTGCCCAAGATCC R: GGTGAGCTTCTGTGCTTCCT	103
*RARβ*	F: TGCTAAAGGTGCAGAACGTG R: ACTGAGCTGGGTGAGATGCT	165
*BMP2*	F: CGTCTGCCTCACGATCAAGT R: ATCCACAAACTCTGGGCTGG	102
*GAPDH*	F: GTCGGTTGTGGATCTGACCT R: CTTGACGAAGTGGTCGTTGA	208

**Table 2:** Summary of sequence quality control and mapping statistics

Sample	Raw reads	Raw base (Gb)	Clean reads	Clean bases (Gb)	Q20 (%)	Q30 (%)	GC content (%)	Clean reads mapped (n, %)	Unique reads mapped (n, %)
ATRA1	91,752,898	13.76	83,880,054	12.58	99.95	97.57	54	69,255,881 (82.57)	60,065,372 (71.61)
ATRA2	86,794,096	13.02	80,062,326	12.01	99.95	97.67	53.5	66,615,388 (83.20)	57,927,309 (72.35)
ATRA3	91,057,522	13.66	82,494,054	12.37	99.94	97.72	52	66,036,586 (80.05)	57,833,351 (70.11)
ATRA4	90,459,066	13.57	80,624,954	12.09	99.93	97.63	54	61,852,876 (76.72)	53,742,774 (66.66)
Control1	93,284,010	13.99	85,127,240	12.77	99.94	98.11	52	68,989,648 (81.04)	60,199,757 (70.72)
Control2	92,224,168	13.83	83,886,126	12.58	99.94	97.74	52.5	67,446,611 (80.40)	59,082,319 (70.43)
Control3	90,298,374	13.54	83,774,634	12.57	99.95	97.82	53	69,825,179 (83.35)	60,383,672 (72.08)
Control4	97,533,500	14.63	88,370,616	13.26	99.94	97.49	53	70,633,259 (79.93)	61,407,588 (69.49)

## Data Availability

Datasets used in this research are available upon reasonable request.

## SUPPLEMENTARY MATERIAL

**Supplementary Table S1.** Differentially expressed lncRNAs after treated with ATRA in porcine granulosa cells

**Supplementary Table S2.** Differentially expressed genes after treated with ATRA in porcine granulosa cells

**Supplementary Table S3.** KEGG analysis of differentially expressed genes

**Supplementary Table S4.** GO and KEGG analysis of the lncRNA target DEGs
