# Supplementary material for: Porcine granulosa cell transcriptomic analyses reveal the differential regulation of lncRNAs and mRNAs in response to all-trans retinoic acid in vitro
Source: Anim Biosci. 2024 Aug 26;38(2):267–77. doi: 10.5713/ab.24.0363 (PMC11725750; doi:10.5713/ab.24.0363)
Supplement: Supplementary file 1 [file ab-24-0363-Supplementary-Table-1.pdf]

Table S1 Differentially expressed lncRNAs after treated with ATRA in porcine granulosa cells

gene_id	FPKM.Sus_ATRA	FPKM.Sus_Contro	log2(fc)	q value	regulation
MSTRG.1752	338.79	14.09	4.59	0.01	up
MSTRG.10023	16.69	1.09	3.94	0.00	up
MSTRG.9089	248.91	33.96	2.87	0.04	up
MSTRG.22226	8.30	1.16	2.83	0.02	up
MSTRG.28520	312.38	50.87	2.62	0.04	up
MSTRG.19921	4.92	0.93	2.40	0.01	up
MSTRG.16899	4.70	0.97	2.27	0.03	up
MSTRG.10112	6.91	1.58	2.13	0.03	up
MSTRG.13000	5.75	1.34	2.10	0.01	up
MSTRG.24293	10.08	2.40	2.07	0.04	up
MSTRG.3188	3.78	0.95	2.00	0.03	up
MSTRG.28505	100.39	27.04	1.89	0.03	up
MSTRG.15239	3.72	1.01	1.88	0.03	up
MSTRG.9318	3.33	0.97	1.78	0.04	up
MSTRG.7027	2.70	0.82	1.72	0.02	up
MSTRG.15532	3.68	1.13	1.70	0.03	up
MSTRG.1318	10.47	3.31	1.66	0.02	up
ENSSSCG00000	263.22	83.93	1.65	0.04	up
MSTRG.12403	3.29	1.06	1.63	0.02	up
MSTRG.8388	2.07	0.68	1.60	0.02	up
MSTRG.6138	5.86	1.98	1.57	0.02	up
MSTRG.1178	13.62	4.60	1.57	0.02	up
MSTRG.7047	3.51	1.19	1.56	0.03	up
MSTRG.6173	1191.08	410.34	1.54	0.04	up
MSTRG.11829	6.16	2.14	1.52	0.02	up
MSTRG.10352	2.57	0.91	1.49	0.03	up
MSTRG.8008	3.72	1.33	1.49	0.02	up
MSTRG.23055	710.94	254.11	1.48	0.04	up
MSTRG.20485	2.73	1.01	1.43	0.03	up
MSTRG.22932	3.56	1.34	1.41	0.03	up
MSTRG.17747	4.51	1.73	1.38	0.03	up
MSTRG.655	2.59	1.02	1.35	0.04	up
MSTRG.26817	2.14	0.84	1.34	0.04	up
MSTRG.15196	2.73	1.08	1.34	0.03	up
MSTRG.13466	4.23	1.67	1.34	0.03	up
MSTRG.4323	2.27	0.92	1.31	0.03	up
MSTRG.28279	2.46	1.01	1.29	0.03	up
MSTRG.27972	2.34	0.97	1.27	0.03	up
MSTRG.10198	3.40	1.42	1.26	0.03	up
MSTRG.25818	2.76	1.18	1.23	0.03	up
MSTRG.19038	70.39	30.07	1.23	0.04	up
MSTRG.6256	11.91	5.11	1.22	0.03	up
MSTRG.21993	61.98	27.45	1.18	0.03	up
MSTRG.25469	2.52	1.14	1.15	0.04	up
MSTRG.407	3.23	1.47	1.13	0.03	up
MSTRG.5679	17.38	8.01	1.12	0.04	up
STRG.21758	3.59	1.66	1.11	0.04	up
MSTRG.8393	1.89	0.88	1.10	0.04	up
MSTRG.2983	4.27	2.00	1.10	0.05	up
MSTRG.26286	10.03	4.70	1.09	0.04	up
ENSSSCG00000	4.40	2.10	1.07	0.04	up

MSTRG.22908	2.58	1.24	1.05	0.05	up
MSTRG.4240	26.75	13.22	1.02	0.04	up
MSTRG.7140	5.46	2.70	1.01	0.04	up
MSTRG.4595	405.73	201.55	1.01	0.04	up
MSTRG.10955	1.04	2.15	-1.05	0.04	down
MSTRG.11967	1.39	2.87	-1.05	0.04	down
MSTRG.13070	1.82	3.83	-1.08	0.04	down
MSTRG.4684	1.71	3.64	-1.09	0.04	down
MSTRG.9538	1.69	3.60	-1.09	0.03	down
MSTRG.20516	3.70	7.93	-1.10	0.04	down
MSTRG.1743	0.62	1.35	-1.12	0.04	down
MSTRG.9216	2.09	4.56	-1.13	0.04	down
MSTRG.25115	0.79	1.72	-1.13	0.04	down
ENSSSCG00000[illegible]	2.61	5.78	-1.15	0.03	down
MSTRG.20115	3.03	6.72	-1.15	0.04	down
MSTRG.10955	1.19	2.65	-1.16	0.04	down
MSTRG.10956	1.77	4.17	-1.24	0.03	down
MSTRG.21404	0.82	1.97	-1.26	0.04	down
MSTRG.19297	2.17	5.26	-1.27	0.03	down
MSTRG.21050	1.40	3.42	-1.29	0.03	down
MSTRG.2877	3.74	10.94	-1.55	0.03	down
MSTRG.28433	0.86	2.59	-1.59	0.04	down
MSTRG.17938	1.30	3.99	-1.61	0.04	down
MSTRG.12191	1.05	3.24	-1.62	0.02	down
MSTRG.27202	1.57	4.91	-1.64	0.02	down
MSTRG.23300	8.40	26.42	-1.65	0.03	down
MSTRG.13160	0.92	3.22	-1.81	0.02	down
MSTRG.2508	1.90	6.73	-1.83	0.02	down
MSTRG.22915	2.22	8.95	-2.01	0.04	down
MSTRG.26895	1.66	7.32	-2.14	0.01	down
MSTRG.15454	1.36	7.33	-2.43	0.02	down
MSTRG.25658	1.08	7.43	-2.79	0.02	down
MSTRG.14041	0.95	11.54	-3.60	0.00	down
MSTRG.26588	1.80	26.41	-3.88	0.00	down
MSTRG.4589	68.42	1504.64	-4.46	0.01	down
