# Supplementary material for: Porcine granulosa cell transcriptomic analyses reveal the differential regulation of lncRNAs and mRNAs in response to all-trans retinoic acid in vitro
Source: Anim Biosci. 2024 Aug 26;38(2):267–77. doi: 10.5713/ab.24.0363 (PMC11725750; doi:10.5713/ab.24.0363)
Supplement: Supplementary file 2 [file ab-24-0363-Supplementary-Table-2.pdf]

Table S2 Differentially expressed genes after treated with ATRA in porcine granulosa cells

gene_id	gene_name	FPKM.Sus_ATRA	FPKM.Sus_Control	log2(fc)	qval	regulation
MSTRG.25392	ENSSSCG00000008981	0.53	3.01	-2.50	0.000206	down
MSTRG.2297	CCDC107	4.85	25.92	-2.42	0.006503	down
MSTRG.10949	RGS7BP	0.65	3.30	-2.36	1.49E-09	down
MSTRG.5603	STAC	0.65	3.01	-2.21	1.36E-08	down
MSTRG.11283	ENSSSCG00000032843	1.14	5.08	-2.16	3.38E-12	down
MSTRG.25736	DKK2	1.89	5.78	-1.61	1.26E-05	down
MSTRG.17332	SHAS2	3.21	9.08	-1.50	2.92E-06	down
MSTRG.18292	ENSSSCG00000035261	2.78	7.75	-1.48	0.006896	down
MSTRG.25349	AMCF-II	3.88	10.58	-1.45	0.000435	down
MSTRG.23414	SAPCD1	0.51	1.38	-1.44	0.004312	down
MSTRG.17816	CYP7B1	1.06	2.84	-1.43	7.02E-06	down
MSTRG.20516	MT2A	11.24	30.10	-1.42	4.68E-05	down
MSTRG.18473	RBM15	1.85	4.76	-1.36	1.69E-05	down
MSTRG.1443	GREM1	1.92	4.91	-1.36	0.016077	down
MSTRG.18033	ADAMTS4	0.75	1.91	-1.35	0.000284	down
MSTRG.14300	GFPT2	0.55	1.36	-1.32	0.000299	down
MSTRG.25118	SGCB	3.26	8.00	-1.30	0.011685	down
MSTRG.28279	APLN	0.97	2.35	-1.28	9.21E-05	down
MSTRG.28112	ARMCX1	0.59	1.43	-1.27	0.0169	down
MSTRG.18293	ENSSSCG00000039615	7.96	18.87	-1.25	0.029858	down
MSTRG.28038	ATRX	1.96	4.64	-1.24	2.04E-05	down
MSTRG.10393	KCNE4	0.74	1.74	-1.23	0.000268	down
MSTRG.25658	PRSS12	3.36	7.88	-1.23	0.001684	down
MSTRG.23017	EEF1E1	2.14	4.90	-1.20	0.003496	down
MSTRG.12424	FAM3C	4.96	11.36	-1.20	0.011054	down
MSTRG.14720	DCP2	3.11	7.08	-1.18	0.00066	down
MSTRG.9578	CASP3	5.94	13.42	-1.18	0.007517	down
MSTRG.8092	LIF	4.54	10.23	-1.17	0.002322	down
MSTRG.6504	PTX3	0.62	1.37	-1.15	0.020709	down
MSTRG.22345	ABHD3	0.95	2.11	-1.14	0.000162	down
MSTRG.13163	FAM111A	2.47	5.35	-1.11	0.027079	down
MSTRG.11960	SNAI1	3.12	6.75	-1.11	2.74E-05	down
MSTRG.27407	LPGAT1	3.83	8.21	-1.10	0.000109	down
MSTRG.24926	KCNIP4	0.56	1.19	-1.09	0.000956	down
MSTRG.8168	ADORA2A	0.62	1.31	-1.09	0.00551	down
MSTRG.8818	ATAD1	2.25	4.74	-1.08	6.42E-05	down
MSTRG.17651	GEM	2.32	4.90	-1.08	0.001127	down
MSTRG.685	TPBG	2.71	5.66	-1.06	0.023	down
MSTRG.28308	FRMD7	0.59	1.21	-1.05	0.003454	down
MSTRG.21695	SLC2A5	2.44	4.97	-1.03	0.000899	down
MSTRG.24558	GPATCH2L	3.64	7.42	-1.03	0.007409	down
MSTRG.4981	CCL2	145.01	293.78	-1.02	0.000118	down
MSTRG.5679	ENSSSCG00000011290	8.49	17.17	-1.02	0.046864	down
MSTRG.28247	ZBTB33	6.36	12.86	-1.02	0.042978	down
MSTRG.255	TNFAIP3	1.94	3.91	-1.01	0.002459	down
MSTRG.25209	GASK1B	2.70	5.44	-1.01	0.011908	down
MSTRG.25350	CXCL2	0.82	1.65	-1.01	0.00335	down
MSTRG.23843	HDGFL3	3.36	6.75	-1.01	0.011164	down
MSTRG.1185	HDC	0.55	1.10	-1.01	0.003343	down
MSTRG.7364	ENSSSCG00000032547	1.29	0.64	1.00	0.039073	up
MSTRG.1429	MEIS2	3.82	1.89	1.01	0.001252	up

MSTRG.21083	ENSSSCG00000003080	5.11	2.52	1.02	0.001737	up
MSTRG.10567	ENSSSCG00000016330	5.62	2.76	1.02	0.027504	up
MSTRG.20579	RRAD	5.03	2.47	1.02	0.045009	up
MSTRG.8422	RTKN2	5.31	2.60	1.03	0.001328	up
MSTRG.7540	LZTS1	5.66	2.76	1.04	0.038354	up
ENSSSCG00000	CYP2E1	31.70	15.43	1.04	0.003782	up
MSTRG.26721	ENSSSCG00000015249	1.49	0.72	1.04	0.00135	up
MSTRG.22015	PTPRU	6.71	3.25	1.05	4.75E-05	up
MSTRG.10351	CYP27A1	1.51	0.73	1.05	0.019671	up
MSTRG.8384	FAM13C	6.22	2.97	1.06	0.000898	up
MSTRG.272	SGK1	22.18	10.61	1.06	1.81E-05	up
MSTRG.22035	TINAGL1	17.03	8.11	1.07	1.52E-05	up
MSTRG.23668	MEA1	5.09	2.42	1.07	0.036511	up
MSTRG.10172	FAM117B	2.70	1.28	1.07	0.007152	up
MSTRG.10308	IGFBP5	168.60	79.99	1.08	0.003834	up
MSTRG.25096	LIMCH1	5.29	2.50	1.08	0.000701	up
MSTRG.8848	ANKRD1	24.13	11.40	1.08	0.017151	up
MSTRG.23734	PLA2G7	3.72	1.75	1.08	6.51E-05	up
MSTRG.3324	MPP7	1.67	0.79	1.09	0.001251	up
MSTRG.1175	CYP19A1	11.23	5.29	1.09	0.036216	up
MSTRG.25400	SHROOM3	8.04	3.78	1.09	0.001009	up
MSTRG.27932	SNX12	6.17	2.89	1.09	0.015437	up
MSTRG.16294	TCF7L1	4.70	2.20	1.09	0.033873	up
MSTRG.17665	ENSSSCG00000032907	1.13	0.52	1.11	0.033873	up
MSTRG.9465	RALB	10.33	4.77	1.11	0.015079	up
MSTRG.10192	CYP20A1	1.22	0.56	1.12	0.021504	up
MSTRG.13746	CCDC124	1.99	0.91	1.13	0.017239	up
MSTRG.17613	SDC2	48.88	22.37	1.13	0.000757	up
MSTRG.407	PLN	3.24	1.48	1.13	0.00125	up
MSTRG.14039	ANGPTL4	85.89	39.14	1.13	1.09E-05	up
MSTRG.27178	SFRP4	114.71	52.24	1.13	1.82E-06	up
MSTRG.4911	PTRH2	1.73	0.78	1.14	0.105872	up
ENSSSCG00000	CYP26B1	1.65	0.74	1.15	0.004043	up
MSTRG.18226	ENSSSCG00000006621	1.83	0.83	1.15	0.001384	up
MSTRG.8112	ENSSSCG00000040755	1.26	0.57	1.15	0.001384	up
MSTRG.11430	BMP2	7.40	3.33	1.15	1.66E-05	up
MSTRG.18351	ENSSSCG00000006719	169.33	76.27	1.15	0.001275	up
MSTRG.25330	NPFFR2	2.32	1.03	1.17	0.001309	up
MSTRG.18218	TUFT1	6.59	2.83	1.22	0.036754	up
MSTRG.11917	MMP9	1.67	0.72	1.22	0.020123	up
MSTRG.28116	ARMCX3	3.99	1.71	1.22	0.040822	up
MSTRG.17728	FABP5	2.72	1.17	1.22	0.003105	up
MSTRG.28126	BEX3	2.76	1.18	1.22	0.046749	up
MSTRG.10695	NPR3	2.18	0.93	1.23	0.001792	up
MSTRG.12678	IGFBP3	14.29	6.05	1.24	2.47E-05	up
MSTRG.22046	TXLNA	14.89	6.26	1.25	0.021067	up
MSTRG.16830	RASGRP3	5.93	2.49	1.25	3E-05	up
MSTRG.13616	ADM	21.85	9.09	1.27	5.91E-05	up
MSTRG.17091	FAM110C	5.11	2.12	1.27	5.13E-05	up
MSTRG.19921	VDR	16.82	6.97	1.27	2.36E-05	up
MSTRG.2521	CDC26	2.03	0.82	1.30	0.010274	up
MSTRG.25468	SH3D19	25.49	10.27	1.31	1.07E-05	up
MSTRG.10169	SUMO1	5.71	2.27	1.33	0.046368	up
MSTRG.18185	NPR1	2.45	0.97	1.33	3.67E-06	up

MSTRG.6138	JAGN1	5.69	2.26	1.33	0.002166	up
MSTRG.8685	ENSSSCG00000010370	9.16	3.62	1.34	0.00019	up
MSTRG.9979	ENSSSCG00000037545	4.62	1.80	1.36	1.96E-06	up
MSTRG.22932	EDN2	3.79	1.45	1.39	7.39E-06	up
MSTRG.8580	SYNPO2L	1.69	0.64	1.39	0.000572	up
MSTRG.13430	KIAA1549L	2.80	1.06	1.40	5.2E-07	up
MSTRG.22506	ENSSSCG00000003753	5.77	2.14	1.43	6.7E-05	up
MSTRG.20282	ZFPM1	8.49	3.15	1.43	0.000654	up
MSTRG.10625	ANKRD33B	5.91	2.19	1.43	0.000205	up
MSTRG.28473	ENSSSCG00000036341	2.34	0.86	1.44	0.042264	up
MSTRG.11827	TGM2	505.98	183.14	1.47	1.67E-05	up
ENSSSCG00000	CYP26B1	6.36	2.27	1.48	0.006207	up
MSTRG.6317	RBP1	6.05	2.10	1.52	0.000559	up
MSTRG.12139	ASB10	1.78	0.61	1.55	7.3E-06	up
MSTRG.10837	HSPB3	1.61	0.55	1.56	9.87E-06	up
MSTRG.4624	CAVIN1	101.21	33.90	1.58	0.001654	up
MSTRG.9162	ABLIM1	3.27	0.99	1.72	1.68E-05	up
MSTRG.17727	FABP4	3.31	0.94	1.82	1.4E-06	up
MSTRG.19091	KRT8	12.13	3.42	1.83	6.93E-09	up
MSTRG.5545	RARB	5.14	1.42	1.86	8.66E-11	up
MSTRG.2509	SLC46A2	2.10	0.52	2.00	6.66E-11	up
MSTRG.3736	HMGB1	12.76	2.58	2.30	0.013189	up
MSTRG.21758	DHRS3	101.39	2.13	5.57	2.07E-85	up
