# Supplementary material for: Porcine granulosa cell transcriptomic analyses reveal the differential regulation of lncRNAs and mRNAs in response to all-trans retinoic acid in vitro
Source: Anim Biosci. 2024 Aug 26;38(2):267–77. doi: 10.5713/ab.24.0363 (PMC11725750; doi:10.5713/ab.24.0363)
Supplement: Supplementary file 3 [file ab-24-0363-Supplementary-Table-3.pdf]

Table S3 KEGG analysis of differentially expressed genes

pathway_id	pathway_name	pvalue	Gene_ID	Gene_name	log2(fc)
ko04657	IL-17 signaling pathway	0.00	MSTRG.25349	AMCF-II	-1.45
ko04657	IL-17 signaling pathway	0.00	MSTRG.25350	ENSSSCG00000008954	-1.01
ko04657	IL-17 signaling pathway	0.00	MSTRG.255	TNFAIP3	-1.01
ko04657	IL-17 signaling pathway	0.00	MSTRG.4981	CCL2	-1.02
ko04657	IL-17 signaling pathway	0.00	MSTRG.6138	JAGN1	1.33
ko04657	IL-17 signaling pathway	0.00	MSTRG.9578	CASP3	-1.18
ko04020	Calcium signaling pathway	0.00	MSTRG.10269	ERBB4	1.33
ko04020	Calcium signaling pathway	0.00	MSTRG.1305	ITPKA	1.34
ko04020	Calcium signaling pathway	0.00	MSTRG.22582	PTGER3	-1.04
ko04020	Calcium signaling pathway	0.00	MSTRG.24909	CD38	2.38
ko04020	Calcium signaling pathway	0.00	MSTRG.407	PLN	1.13
ko04020	Calcium signaling pathway	0.00	MSTRG.5032	NOS2	1.64
ko04020	Calcium signaling pathway	0.00	MSTRG.8168	ADORA2A	-1.09
ko04020	Calcium signaling pathway	0.00	MSTRG.8838	HTR7	-1.13
ko04668	TNF signaling pathway	0.00	MSTRG.25350	ENSSSCG00000008954	-1.01
ko04668	TNF signaling pathway	0.00	MSTRG.255	TNFAIP3	-1.01
ko04668	TNF signaling pathway	0.00	MSTRG.4981	CCL2	-1.02
ko04668	TNF signaling pathway	0.00	MSTRG.8092	LIF	-1.17
ko04668	TNF signaling pathway	0.00	MSTRG.9578	CASP3	-1.18
ko04060	Cytokine-cytokine receptor interaction	0.00	ENSSSCG00000020906	TNFSF10	-1.34
ko04060	Cytokine-cytokine receptor interaction	0.00	ENSSSCG00000022370	TNFSF9	-1.13
ko04060	Cytokine-cytokine receptor interaction	0.00	MSTRG.11430	BMP2	1.15
ko04060	Cytokine-cytokine receptor interaction	0.00	MSTRG.25349	AMCF-II	-1.45
ko04060	Cytokine-cytokine receptor interaction	0.00	MSTRG.25350	ENSSSCG00000008954	-1.01
ko04060	Cytokine-cytokine receptor interaction	0.00	MSTRG.4981	CCL2	-1.02
ko04060	Cytokine-cytokine receptor interaction	0.00	MSTRG.6138	JAGN1	1.33
ko04060	Cytokine-cytokine receptor interaction	0.00	MSTRG.8092	LIF	-1.17
ko04923	Regulation of lipolysis in adipocytes	0.00	MSTRG.17727	FABP4	1.82
ko04923	Regulation of lipolysis in adipocytes	0.00	MSTRG.18185	NPR1	1.33
ko04923	Regulation of lipolysis in adipocytes	0.00	MSTRG.22582	PTGER3	-1.04
ko05200	Pathways in cancer	0.00	ENSSSCG00000030095	ZBTB16	1.47
ko05200	Pathways in cancer	0.00	ENSSSCG00000031924	NKX3-1	1.46
ko05200	Pathways in cancer	0.00	MSTRG.11430	BMP2	1.15
ko05200	Pathways in cancer	0.00	MSTRG.13227	F2	1.12
ko05200	Pathways in cancer	0.00	MSTRG.16294	TCF7L1	1.09
ko05200	Pathways in cancer	0.00	MSTRG.16830	RASGRP3	1.25
ko05200	Pathways in cancer	0.00	MSTRG.22582	PTGER3	-1.04
ko05200	Pathways in cancer	0.00	MSTRG.5032	NOS2	1.64
ko05200	Pathways in cancer	0.00	MSTRG.5545	RARB	1.86
ko05200	Pathways in cancer	0.00	MSTRG.9465	RALB	1.11
ko05200	Pathways in cancer	0.00	MSTRG.9578	CASP3	-1.18
ko04024	cAMP signaling pathway	0.00	MSTRG.18185	NPR1	1.33

ko04024	cAMP signaling pathway	0.00	MSTRG.22582	PTGER3	-1.04
ko04024	cAMP signaling pathway	0.00	MSTRG.22633	ENSSSCG00000040903	-1.17
ko04024	cAMP signaling pathway	0.00	MSTRG.22932	EDN2	1.39
ko04024	cAMP signaling pathway	0.00	MSTRG.407	PLN	1.13
ko04024	cAMP signaling pathway	0.00	MSTRG.8168	ADORA2A	-1.09
ko04080	Neuroactive ligand-receptor interaction	0.00	MSTRG.13227	F2	1.12
ko04080	Neuroactive ligand-receptor interaction	0.00	MSTRG.13616	ADM	1.27
ko04080	Neuroactive ligand-receptor interaction	0.00	MSTRG.22582	PTGER3	-1.04
ko04080	Neuroactive ligand-receptor interaction	0.00	MSTRG.22932	EDN2	1.39
ko04080	Neuroactive ligand-receptor interaction	0.00	MSTRG.23767	CHRNB4	1.33
ko04080	Neuroactive ligand-receptor interaction	0.00	MSTRG.25330	NPFFR2	1.17
ko04080	Neuroactive ligand-receptor interaction	0.00	MSTRG.8168	ADORA2A	-1.09
ko04080	Neuroactive ligand-receptor interaction	0.00	MSTRG.8838	HTR7	-1.13
ko04928	Parathyroid hormone synthesis, secretion and action	0.00	ENSSSCG00000014852	ARRB1	-1.40
ko04928	Parathyroid hormone synthesis, secretion and action	0.00	MSTRG.19557	PTHLH	-1.68
ko04928	Parathyroid hormone synthesis, secretion and action	0.00	MSTRG.19921	VDR	1.27
ko04928	Parathyroid hormone synthesis, secretion and action	0.00	MSTRG.22633	ENSSSCG00000040903	-1.17
ko00760	Nicotinate and nicotinamide metabolism	0.00	MSTRG.2237	NMRK1	-1.07
ko00760	Nicotinate and nicotinamide metabolism	0.00	MSTRG.24909	CD38	2.38
ko05133	Pertussis	0.00	MSTRG.25349	AMCF-II	-1.45
ko05133	Pertussis	0.00	MSTRG.5032	NOS2	1.64
ko05133	Pertussis	0.00	MSTRG.9578	CASP3	-1.18
ko04710	Circadian rhythm	0.00	MSTRG.19587	BHLHE41	-1.14
ko04710	Circadian rhythm	0.00	MSTRG.6109	BHLHE40	1.04
ko03320	PPAR signaling pathway	0.01	MSTRG.14039	ANGPTL4	1.13
ko03320	PPAR signaling pathway	0.01	MSTRG.17727	FABP4	1.82
ko03320	PPAR signaling pathway	0.01	MSTRG.17728	FABP5	1.22
ko00250	Alanine, aspartate and glutamate metabolism	0.01	MSTRG.14300	GFPT2	-1.32
ko00250	Alanine, aspartate and glutamate metabolism	0.01	MSTRG.24803	NAT8L	1.02
ko04270	Vascular smooth muscle contraction	0.01	MSTRG.13616	ADM	1.27
ko04270	Vascular smooth muscle contraction	0.01	MSTRG.18185	NPR1	1.33
ko04270	Vascular smooth muscle contraction	0.01	MSTRG.22932	EDN2	1.39

ko04270	Vascular smooth muscle contraction	0.01	MSTRG.8168	ADORA2A	-1.09
ko04978	Mineral absorption	0.01	MSTRG.19921	VDR	1.27
ko04978	Mineral absorption	0.01	MSTRG.20516	MT2A	-1.42
ko04217	Necroptosis	0.01	ENSSSCG00000020906	TNFSF10	-1.34
ko04217	Necroptosis	0.01	MSTRG.18293	ENSSSCG00000039615	-1.25
ko04217	Necroptosis	0.01	MSTRG.255	TNFAIP3	-1.01
ko04217	Necroptosis	0.01	MSTRG.3736	HMGB1	2.30
ko05222	Small cell lung cancer	0.01	MSTRG.5032	NOS2	1.64
ko05222	Small cell lung cancer	0.01	MSTRG.5545	RARB	1.86
ko05222	Small cell lung cancer	0.01	MSTRG.9578	CASP3	-1.18
ko05210	Colorectal cancer	0.01	MSTRG.16294	TCF7L1	1.09
ko05210	Colorectal cancer	0.01	MSTRG.9465	RALB	1.11
ko05210	Colorectal cancer	0.01	MSTRG.9578	CASP3	-1.18
ko00140	Steroid hormone biosynthesis	0.01	MSTRG.17816	CYP7B1	-1.43
ko00140	Steroid hormone biosynthesis	0.01	MSTRG.18351	ENSSSCG00000006719	1.15
ko05144	Malaria	0.01	MSTRG.17613	ENSSSCG00000038665	1.13
ko05144	Malaria	0.01	MSTRG.4981	CCL2	-1.02
ko00330	Arginine and proline metabolism	0.01	MSTRG.21109	CKM	-1.33
ko00330	Arginine and proline metabolism	0.01	MSTRG.5032	NOS2	1.64
ko05134	Legionellosis	0.01	MSTRG.25350	ENSSSCG00000008954	-1.01
ko05134	Legionellosis	0.01	MSTRG.9578	CASP3	-1.18
ko00830	Retinol metabolism	0.01	ENSSSCG00000008311	CYP26B1	2.86
ko00830	Retinol metabolism	0.01	MSTRG.21758	DHRS3	5.57
ko05416	Viral myocarditis	0.01	MSTRG.25118	SGCB	-1.30
ko05416	Viral myocarditis	0.01	MSTRG.9578	CASP3	-1.18
ko04062	Chemokine signaling	0.01	ENSSSCG00000014852	ARRB1	-1.40
ko04062	Chemokine signaling	0.01	MSTRG.25349	AMCF-II	-1.45
ko04062	Chemokine signaling	0.01	MSTRG.25350	ENSSSCG00000008954	-1.01
ko04062	Chemokine signaling	0.01	MSTRG.4981	CCL2	-1.02
ko05217	Basal cell carcinoma	0.02	MSTRG.11430	BMP2	1.15
ko05217	Basal cell carcinoma	0.02	MSTRG.16294	TCF7L1	1.09
ko05221	Acute myeloid leukemia	0.02	ENSSSCG00000030095	ZBTB16	1.47
ko05221	Acute myeloid leukemia	0.02	MSTRG.16294	TCF7L1	1.09
ko00120	Primary bile acid	0.02	MSTRG.17816	CYP7B1	-1.43
ko04115	p53 signaling pathway	0.02	MSTRG.12678	IGFBP3	1.24
ko04115	p53 signaling pathway	0.02	MSTRG.9578	CASP3	-1.18
ko04924	Renin secretion	0.02	MSTRG.18185	NPR1	1.33
ko04924	Renin secretion	0.02	MSTRG.22932	EDN2	1.39
ko05412	Arrhythmogenic right ventricular cardiomyopathy (ARVC)	0.02	MSTRG.16294	TCF7L1	1.09
ko05412	Arrhythmogenic right ventricular cardiomyopathy (ARVC)	0.02	MSTRG.25118	SGCB	-1.30
ko04210	Apoptosis	0.02	ENSSSCG00000020906	TNFSF10	-1.34
ko04210	Apoptosis	0.02	MSTRG.8211	ENSSSCG00000010103	-4.60
ko04210	Apoptosis	0.02	MSTRG.9578	CASP3	-1.18
ko05034	Alcoholism	0.02	MSTRG.18292	ENSSSCG00000035261	-1.48
ko05034	Alcoholism	0.02	MSTRG.18293	ENSSSCG00000039615	-1.25
ko05034	Alcoholism	0.02	MSTRG.8168	ADORA2A	-1.09
ko04520	Adherens junction	0.02	MSTRG.11960	SNAI1	-1.11

ko04520	Adherens junction	0.02	MSTRG.16294	TCF7L1	1.09
ko05132	Salmonella infection	0.02	MSTRG.25350	ENSSSCG00000008954	-1.01
ko05132	Salmonella infection	0.02	MSTRG.5032	NOS2	1.64
ko00220	Arginine biosynthesis	0.02	MSTRG.5032	NOS2	1.64
ko04621	NOD-like receptor signaling pathway	0.02	MSTRG.25350	ENSSSCG00000008954	-1.01
ko04621	NOD-like receptor signaling pathway	0.02	MSTRG.255	TNFAIP3	-1.01
ko04621	NOD-like receptor signaling pathway	0.02	MSTRG.4981	CCL2	-1.02
ko05322	Systemic lupus erythematosus	0.02	MSTRG.18292	ENSSSCG00000035261	-1.48
ko05322	Systemic lupus erythematosus	0.02	MSTRG.18293	ENSSSCG00000039615	-1.25
ko04010	MAPK signaling pathway	0.02	ENSSSCG00000012774	DUSP9	-1.22
ko04010	MAPK signaling pathway	0.02	ENSSSCG00000014852	ARRB1	-1.40
ko04010	MAPK signaling pathway	0.02	MSTRG.10269	ERBB4	1.33
ko04010	MAPK signaling pathway	0.02	MSTRG.16830	RASGRP3	1.25
ko04010	MAPK signaling pathway	0.02	MSTRG.9578	CASP3	-1.18
ko00340	Histidine metabolism	0.02	MSTRG.1185	HDC	-1.01
ko04977	Vitamin digestion and absorption	0.02	MSTRG.10474	SLC19A3	1.66
ko05323	Rheumatoid arthritis	0.02	MSTRG.25349	AMCF-II	-1.45
ko05323	Rheumatoid arthritis	0.02	MSTRG.4981	CCL2	-1.02
ko05414	Dilated cardiomyopathy (DCM)	0.02	MSTRG.25118	SGCB	-1.30
ko05414	Dilated cardiomyopathy (DCM)	0.02	MSTRG.407	PLN	1.13
ko04310	Wnt signaling pathway	0.03	MSTRG.16294	TCF7L1	1.09
ko04310	Wnt signaling pathway	0.03	MSTRG.25736	DKK2	-1.61
ko04310	Wnt signaling pathway	0.03	MSTRG.27178	SFRP4	1.13
ko05152	Tuberculosis	0.03	MSTRG.19921	VDR	1.27
ko05152	Tuberculosis	0.03	MSTRG.5032	NOS2	1.64
ko05152	Tuberculosis	0.03	MSTRG.9578	CASP3	-1.18
ko05146	Amoebiasis	0.03	MSTRG.5032	NOS2	1.64
ko05146	Amoebiasis	0.03	MSTRG.9578	CASP3	-1.18
ko05032	Morphine addiction	0.03	ENSSSCG00000014852	ARRB1	-1.40
ko05032	Morphine addiction	0.03	MSTRG.22633	ENSSSCG00000040903	-1.17
ko04530	Tight junction	0.03	ENSSSCG00000040904	CLDN1	-1.33
ko04530	Tight junction	0.03	MSTRG.18226	ENSSSCG00000006621	1.15
ko04530	Tight junction	0.03	MSTRG.8211	ENSSSCG00000010103	-4.60
ko04350	TGF-beta signaling pathway	0.03	MSTRG.11430	BMP2	1.15
ko04350	TGF-beta signaling pathway	0.03	MSTRG.1443	GREM1	-1.36
ko04925	Aldosterone synthesis and secretion	0.03	MSTRG.18185	NPR1	1.33
ko04925	Aldosterone synthesis and secretion	0.03	MSTRG.18351	ENSSSCG00000006719	1.15
ko05215	Prostate cancer	0.03	ENSSSCG00000031924	NKX3-1	1.46
ko05215	Prostate cancer	0.03	MSTRG.16294	TCF7L1	1.09
ko05202	Transcriptional misregulation in cancer	0.03	ENSSSCG00000030095	ZBTB16	1.47
ko05202	Transcriptional misregulation in cancer	0.03	MSTRG.12678	IGFBP3	1.24
ko05202	Transcriptional misregulation in cancer	0.03	MSTRG.18292	ENSSSCG00000035261	-1.48

ko04215	Apoptosis - multiple species	0.03	MSTRG.9578	CASP3	-1.18
ko04933	AGE-RAGE signaling pathway in diabetic complications	0.03	MSTRG.4981	CCL2	-1.02
ko04933	AGE-RAGE signaling pathway in diabetic complications	0.03	MSTRG.9578	CASP3	-1.18
ko05145	Toxoplasmosis	0.03	MSTRG.5032	NOS2	1.64
ko05145	Toxoplasmosis	0.03	MSTRG.9578	CASP3	-1.18
ko03410	Base excision repair	0.03	MSTRG.3736	HMGB1	2.30
ko04650	Natural killer cell mediated cytotoxicity	0.03	ENSSSCG00000020906	TNFSF10	-1.34
ko04650	Natural killer cell mediated cytotoxicity	0.03	MSTRG.9578	CASP3	-1.18
ko05142	Chagas disease (American trypanosomiasis)	0.03	MSTRG.4981	CCL2	-1.02
ko05142	Chagas disease (American trypanosomiasis)	0.03	MSTRG.5032	NOS2	1.64
ko04960	Aldosterone-regulated sodium reabsorption	0.03	MSTRG.272	SGK1	1.06
ko04726	Serotonergic synapse	0.03	MSTRG.8838	HTR7	-1.13
ko04726	Serotonergic synapse	0.03	MSTRG.9578	CASP3	-1.18
ko05216	Thyroid cancer	0.03	MSTRG.16294	TCF7L1	1.09
ko04725	Cholinergic synapse	0.04	MSTRG.23767	CHRNB4	1.33
ko04725	Cholinergic synapse	0.04	MSTRG.443	KCNQ5	-1.09
ko04973	Carbohydrate digestion and absorption	0.04	MSTRG.21695	SLC2A5	-1.03
ko05205	Proteoglycans in cancer	0.04	MSTRG.10269	ERBB4	1.33
ko05205	Proteoglycans in cancer	0.04	MSTRG.17613	ENSSSCG00000038665	1.13
ko05205	Proteoglycans in cancer	0.04	MSTRG.9578	CASP3	-1.18
ko04962	Vasopressin-regulated water reabsorption	0.04	ENSSSCG00000040513	AQP3	-1.18
ko04961	Endocrine and other factor-regulated calcium reabsorption	0.04	MSTRG.19921	VDR	1.27
ko02010	ABC transporters	0.04	MSTRG.18632	ABCA4	1.39
ko00565	Ether lipid metabolism	0.04	MSTRG.23734	PLA2G7	1.08
ko04913	Ovarian steroidogenesis	0.04	MSTRG.18351	ENSSSCG00000006719	1.15
ko04015	Rap1 signaling pathway	0.04	MSTRG.16830	RASGRP3	1.25
ko04015	Rap1 signaling pathway	0.04	MSTRG.8168	ADORA2A	-1.09
ko04015	Rap1 signaling pathway	0.04	MSTRG.9465	RALB	1.11
ko04340	Hedgehog signaling pathway	0.04	ENSSSCG00000014852	ARRB1	-1.40
ko04979	Cholesterol metabolism	0.04	MSTRG.14039	ANGPTL4	1.13
ko05162	Measles	0.04	MSTRG.255	TNFAIP3	-1.01
ko05162	Measles	0.04	MSTRG.9578	CASP3	-1.18
ko05418	Fluid shear stress and atherosclerosis	0.04	MSTRG.17613	ENSSSCG00000038665	1.13
ko05418	Fluid shear stress and atherosclerosis	0.04	MSTRG.4981	CCL2	-1.02
ko04926	Relaxin signaling pathway	0.04	ENSSSCG00000014852	ARRB1	-1.40
ko04926	Relaxin signaling pathway	0.04	MSTRG.5032	NOS2	1.64
ko05014	Amyotrophic lateral sclerosis (ALS)	0.04	MSTRG.9578	CASP3	-1.18
ko05163	Human cytomegalovirus infection	0.04	MSTRG.22582	PTGER3	-1.04

ko05163	Human cytomegalovirus infection	0.04	MSTRG.4981	CCL2	-1.02
ko05163	Human cytomegalovirus infection	0.04	MSTRG.9578	CASP3	-1.18
ko00230	Purine metabolism	0.04	MSTRG.18185	NPR1	1.33
ko00230	Purine metabolism	0.04	MSTRG.22633	ENSSSCG00000040903	-1.17
ko00520	Amino sugar and nucleotide sugar metabolism	0.04	MSTRG.14300	GFPT2	-1.32
ko04550	Signaling pathways regulating pluripotency of	0.04	ENSSSCG00000012774	DUSP9	-1.22
ko04550	Signaling pathways regulating pluripotency of	0.04	MSTRG.8092	LIF	-1.17
ko04068	FoxO signaling pathway	0.04	ENSSSCG00000020906	TNFSF10	-1.34
ko04068	FoxO signaling pathway	0.04	MSTRG.272	SGK1	1.06
ko04514	Cell adhesion molecules (CAMs)	0.04	ENSSSCG00000040904	CLDN1	-1.33
ko04514	Cell adhesion molecules (CAMs)	0.04	MSTRG.17613	ENSSSCG00000038665	1.13
ko04014	Ras signaling pathway	0.00	MSTRG.16830	RASGRP3	1.25
ko04014	Ras signaling pathway	0.00	MSTRG.8838	HTR7	-1.13
ko04014	Ras signaling pathway	0.00	MSTRG.9465	RALB	1.11
ko05160	Hepatitis C	0.00	ENSSSCG00000040904	CLDN1	-1.33
ko05160	Hepatitis C	0.00	MSTRG.9578	CASP3	-1.18
ko05213	Endometrial cancer	0.00	MSTRG.16294	TCF7L1	1.09
ko05012	Parkinson disease	0.00	MSTRG.8168	ADORA2A	-1.09
ko05012	Parkinson disease	0.00	MSTRG.9578	CASP3	-1.18
ko05164	Influenza A	0.01	ENSSSCG00000020906	TNFSF10	-1.34
ko05164	Influenza A	0.01	MSTRG.4981	CCL2	-1.02
ko04072	Phospholipase D signaling pathway	0.01	MSTRG.13227	F2	1.12
ko04072	Phospholipase D signaling pathway	0.01	MSTRG.9465	RALB	1.11
ko04921	Oxytocin signaling pathway	0.01	MSTRG.18185	NPR1	1.33
ko04921	Oxytocin signaling pathway	0.01	MSTRG.24909	CD38	2.38
ko05226	Gastric cancer	0.01	MSTRG.16294	TCF7L1	1.09
ko05226	Gastric cancer	0.01	MSTRG.5545	RARB	1.86
ko05140	Leishmaniasis	0.01	MSTRG.5032	NOS2	1.64
ko04927	Cortisol synthesis and secretion	0.01	MSTRG.18351	ENSSSCG00000006719	1.15
ko05223	Non-small cell lung cancer	0.01	MSTRG.5545	RARB	1.86
ko04934	Cushing syndrome	0.01	MSTRG.16294	TCF7L1	1.09
ko04934	Cushing syndrome	0.01	MSTRG.18351	ENSSSCG00000006719	1.15
ko01524	Platinum drug resistance	0.01	MSTRG.9578	CASP3	-1.18
ko04390	Hippo signaling pathway	0.01	MSTRG.11430	BMP2	1.15
ko04390	Hippo signaling pathway	0.01	MSTRG.16294	TCF7L1	1.09
ko04662	B cell receptor signaling pathway	0.01	MSTRG.16830	RASGRP3	1.25
ko04742	Taste transduction	0.01	ENSSSCG00000015911	SCN3A	-1.26
ko04976	Bile secretion	0.01	MSTRG.20003	NR1H4	-1.25
ko04022	cGMP-PKG signaling pathway	0.01	MSTRG.18185	NPR1	1.33
ko04022	cGMP-PKG signaling pathway	0.01	MSTRG.407	PLN	1.13
ko04970	Salivary secretion	0.01	MSTRG.24909	CD38	2.38

ko00562	Inositol phosphate metabolism	0.01	MSTRG.1305	ITPKA	1.34
ko04610	Complement and coagulation cascades	0.01	MSTRG.13227	F2	1.12
ko05212	Pancreatic cancer	0.01	MSTRG.9465	RALB	1.11
ko05167	Kaposi sarcoma-associated herpesvirus infection	0.01	MSTRG.25350	ENSSSCG00000008954	-1.01
ko05167	Kaposi sarcoma-associated herpesvirus infection	0.01	MSTRG.9578	CASP3	-1.18
ko04512	ECM-receptor interaction	0.01	ENSSSCG00000002262	SV2B	-1.85
ko03018	RNA degradation	0.01	MSTRG.14720	DCP2	-1.18
ko04974	Protein digestion and absorption	0.01	MSTRG.9119	COL17A1	-1.39
ko05410	Hypertrophic cardiomyopathy (HCM)	0.01	MSTRG.25118	SGCB	-1.30
ko05169	Epstein-Barr virus infection	0.01	MSTRG.255	TNFAIP3	-1.01
ko05169	Epstein-Barr virus infection	0.01	MSTRG.9578	CASP3	-1.18
ko04012	ErbB signaling pathway	0.01	MSTRG.10269	ERBB4	1.33
ko04640	Hematopoietic cell lineage	0.01	MSTRG.24909	CD38	2.38
ko04146	Peroxisome	0.01	MSTRG.5032	NOS2	1.64
ko04540	Gap junction	0.01	MSTRG.8211	ENSSSCG00000010103	-4.60
ko04914	Progesterone-mediated oocyte maturation	0.01	MSTRG.2521	CDC26	1.30
ko04064	NF-kappa B signaling pathway	0.01	MSTRG.255	TNFAIP3	-1.01
ko05016	Huntington disease	0.01	MSTRG.11827	TGM2	1.47
ko05016	Huntington disease	0.01	MSTRG.9578	CASP3	-1.18
ko04972	Pancreatic secretion	0.01	MSTRG.24909	CD38	2.38
ko00564	Glycerophospholipid metabolism	0.01	MSTRG.27407	LPGAT1	-1.10
ko04066	HIF-1 signaling pathway	0.01	MSTRG.5032	NOS2	1.64
ko04070	Phosphatidylinositol signaling system	0.01	MSTRG.1305	ITPKA	1.34
ko04916	Melanogenesis	0.01	MSTRG.16294	TCF7L1	1.09
ko04670	Leukocyte transendothelial migration	0.01	ENSSSCG00000040904	CLDN1	-1.33
ko04931	Insulin resistance	0.01	MSTRG.14300	GFPT2	-1.32
ko04114	Oocyte meiosis	0.01	MSTRG.2521	CDC26	1.30
ko04919	Thyroid hormone signaling pathway	0.01	MSTRG.407	PLN	1.13
ko04110	Cell cycle	0.01	MSTRG.2521	CDC26	1.30
ko04611	Platelet activation	0.01	MSTRG.13227	F2	1.12
ko04144	Endocytosis	0.01	ENSSSCG00000014852	ARRB1	-1.40
ko04144	Endocytosis	0.01	MSTRG.27932	SNX12	1.09
ko04915	Estrogen signaling pathway	0.01	MSTRG.4657	KRT14	3.25
ko04140	Autophagy - animal	0.01	MSTRG.3736	HMGB1	2.30
ko04371	Apelin signaling pathway	0.01	MSTRG.5032	NOS2	1.64
ko04120	Ubiquitin mediated proteolysis	0.01	MSTRG.2521	CDC26	1.30
ko04145	Phagosome	0.01	MSTRG.8211	ENSSSCG00000010103	-4.60
ko04261	Adrenergic signaling in cardiomyocytes	0.01	MSTRG.407	PLN	1.13
ko05206	MicroRNAs in cancer	0.01	MSTRG.9578	CASP3	-1.18
ko05224	Breast cancer	0.01	MSTRG.16294	TCF7L1	1.09
ko04218	Cellular senescence	0.01	MSTRG.12678	IGFBP3	1.24

ko04932	Non-alcoholic fatty liver disease (NAFLD)	0.01	MSTRG.9578	CASP3	-1.18
ko04630	Jak-STAT signaling pathway	0.01	MSTRG.8092	LIF	-1.17
ko04150	mTOR signaling pathway	0.01	MSTRG.272	SGK1	1.06
ko05161	Hepatitis B	0.01	MSTRG.9578	CASP3	-1.18
ko05225	Hepatocellular carcinoma	0.01	MSTRG.16294	TCF7L1	1.09
ko05203	Viral carcinogenesis	0.01	MSTRG.9578	CASP3	-1.18
ko05010	Alzheimer disease	0.01	MSTRG.9578	CASP3	-1.18
ko05165	Human papillomavirus infection	0.01	MSTRG.16294	TCF7L1	1.09
ko05165	Human papillomavirus infection	0.01	MSTRG.9578	CASP3	-1.18
ko04360	Axon guidance	0.01	MSTRG.9162	ABLIM1	1.72
ko05168	Herpes simplex virus 1 infection	0.01	MSTRG.4981	CCL2	-1.02
ko05168	Herpes simplex virus 1 infection	0.01	MSTRG.9578	CASP3	-1.18
ko05170	Human immunodeficiency virus 1 infection	0.01	MSTRG.9578	CASP3	-1.18
ko04151	PI3K-Akt signaling pathway	0.01	MSTRG.10269	ERBB4	1.33
ko04151	PI3K-Akt signaling pathway	0.01	MSTRG.272	SGK1	1.06
ko04810	Regulation of actin cytoskeleton	0.01	MSTRG.13227	F2	1.12
ko05166	Human T-cell leukemia virus 1 infection	0.01	MSTRG.2521	CDC26	1.30
ko04714	Thermogenesis	0.01	MSTRG.18185	NPR1	1.33
ko04740	Olfactory transduction	0.01	ENSSSCG00000014852	ARRB1	-1.40
