# Supplementary material for: Porcine granulosa cell transcriptomic analyses reveal the differential regulation of lncRNAs and mRNAs in response to all-trans retinoic acid in vitro
Source: Anim Biosci. 2024 Aug 26;38(2):267–77. doi: 10.5713/ab.24.0363 (PMC11725750; doi:10.5713/ab.24.0363)
Supplement: Supplementary file 4 [file ab-24-0363-Supplementary-Table-4.pdf]

Table S4 GO and KEGG analysis of the lncRNA target DEGs

gene_name	GO	KEGG	lncRNA_id
CYP20A1	GO:0005506(iron ion binding);GO:0005515(protein binding);GO:0016705(oxidoreductase activity, acting on paired donors, with incorporation or reduction of molecular oxygen);GO:0020037(heme binding);GO:0055114(oxidation-reduction process)	NA	MSTRG.10198
CYP27A1	GO:0004497(monooxygenase activity);GO:0005506(iron ion binding);GO:0005739(mitochondrion);GO:0006699(bile acid biosynthetic process);GO:0006707(cholesterol catabolic process);GO:0016491(oxidoreductase activity);GO:0016705(oxidoreductase activity, acting on paired donors, with incorporation or reduction of molecular oxygen);GO:0020037(heme binding);GO:0030343(vitamin D3 25-hydroxylase activity);GO:0031073(cholesterol 26-hydroxylase activity);GO:0036378(calcitriol biosynthetic process from calciol);GO:0046872(metal ion binding);GO:0047749(cholestanetriol 26-monooxygenase activity);GO:0055114(oxidation-reduction process)	00120(Primary bile acid biosynthesis);03320(PPAR signaling pathway);04979(Cholesterol metabolism)	MSTRG.10352
CYP19A1	GO:0002677(negative regulation of chronic inflammatory response);GO:0004497(monooxygenase activity);GO:0005506(iron ion binding);GO:0005783(endoplasmic reticulum);GO:0006710(androgen catabolic process);GO:0008209(androgen metabolic process);GO:0008585(female gonad development);GO:0010760(negative regulation of macrophage chemotaxis);GO:0016020(membrane);GO:0016021(integral component of membrane);GO:0016491(oxidoreductase activity);GO:0016705(oxidoreductase activity, acting on paired donors, with incorporation or reduction of molecular oxygen);GO:0016712(oxidoreductase activity, acting on paired donors, with incorporation or reduction of molecular oxygen, reduced flavin or flavoprotein as one donor, and incorporation of one atom of oxygen);GO:0020037(heme binding);GO:0030540(female genitalia development);GO:0030879(mammary gland development);GO:0046872(metal ion binding);GO:0055114(oxidation-reduction process);GO:0060065(uterus development);GO:0060736(prostate gland growth);GO:0061370(testosterone biosynthetic process);GO:0070330(aromatase activity);GO:2000866(positive regulation of estradiol secretion)	00140(Steroid hormone biosynthesis);04913(Ovarian steroidogenesis)	MSTRG.1178

ANGPTL4	GO:0001525(angiogenesis);GO:0004857(enzyme inhibitor activity);GO:0005576(extracellular region);GO:0005615(extracellular space);GO:0007275(multicellular organism development);GO:0030154(cell differentiation);GO:0042802(identical protein binding);GO:0043066(negative regulation of apoptotic process);GO:0051005(negative regulation of lipoprotein lipase activity);GO:0070328(triglyceride homeostasis);GO:2000352(negative regulation of endothelial cell apoptotic process)	03320(PPAR signaling pathway);04979(Cholesterol metabolism)	MSTRG.14041
VDR	GO:0000122(negative regulation of transcription by RNA polymerase II);GO:0000902(cell morphogenesis);GO:0000976(transcription regulatory region sequence-specific DNA binding);GO:0000977(RNA polymerase II regulatory region sequence-specific DNA binding);GO:0000981(DNA-binding transcription factor activity, RNA polymerase II-specific);GO:0001501(skeletal system development);GO:0003677(DNA binding);GO:0003700(DNA-binding transcription factor activity);GO:0003707(steroid hormone receptor activity);GO:0004879(nuclear receptor activity);GO:0005499(vitamin D binding);GO:0005634(nucleus);GO:0005654(nucleoplasm);GO:0005737(cytoplasm);GO:0006355(regulation of transcription, DNA-templated);GO:0006816(calcium ion transport);GO:0006874(cellular calcium ion homeostasis);GO:0007275(multicellular organism development);GO:0007595(lactation);GO:0008134(transcription factor binding);GO:0008270(zinc ion binding);GO:0008285(negative regulation of cell population proliferation);GO:0009887(animal organ morphogenesis);GO:0010628(positive regulation of gene expression);GO:0010839(negative regulation of keratinocyte proliferation);GO:0010980(positive regulation of vitamin D 24-hydroxylase activity);GO:0030154(cell differentiation);GO:0030374(nuclear receptor transcription coactivator activity);GO:0030522(intracellular receptor signaling pathway);GO:0038023(signaling receptor activity);GO:0038183(bile acid signaling pathway);GO:0038186(lithocholic acid receptor activity);GO:0043235(receptor complex);GO:0043401(steroid hormone mediated signaling pathway);GO:0043565(sequence-specific DNA binding);GO:0045618(positive regulation of keratinocyte differentiation);GO:0045892(negative regulation of transcription, DNA-templated);GO:0045944(positive regulation of transcription by RNA polymerase II);GO:0046697(decidualization);GO:0046872(metal ion binding);GO:0046965(retinoid X receptor binding);GO:0050892(intestinal absorption);GO:0060058(positive regulation of apoptotic process involved in mammary gland involution);GO:0060558(regulation of calcidiol 1-monooxygenase activity);GO:0060745(mammary gland branching involved in	04928(Parathyroid hormone synthesis, secretion and action);04961(Endocrine and other factor-regulated calcium reabsorption);04978(Mineral absorption);05152(Tuberculosis)	MSTRG.19921

MT2A	GO:0005634(nucleus);GO:0005737(cytoplasm);GO:0006882(cellular zinc ion homeostasis);GO:0007263(nitric oxide mediated signal transduction);GO:0008144(drug binding);GO:0008270(zinc ion binding);GO:0009617(response to bacterium);GO:0010273(detoxification of copper ion);GO:0035690(cellular response to drug);GO:0036016(cellular response to interleukin-3);GO:0036018(cellular response to erythropoietin);GO:0045926(negative regulation of growth);GO:0046872(metal ion binding);GO:0048471(perinuclear region of cytoplasm);GO:0071276(cellular response to cadmium ion);GO:0071280(cellular response to copper ion);GO:0071294(cellular response to zinc ion)	04978(Mineral absorption)	MSTRG.20516
DHRS3	GO:0001523(retinoid metabolic process);GO:0003151(outflow tract morphogenesis);GO:0004745(retinol dehydrogenase activity);GO:0005811(lipid droplet);GO:0016020(membrane);GO:0016021(integral component of membrane);GO:0030278(regulation of ossification);GO:0042572(retinol metabolic process);GO:0048385(regulation of retinoic acid receptor signaling pathway);GO:0048387(negative regulation of retinoic acid receptor signaling pathway);GO:0055114(oxidation-reduction process);GO:0060021(roof of mouth development);GO:0060349(bone morphogenesis);GO:0060411(cardiac septum morphogenesis)	00830(Retinol metabolism)	MSTRG.21756
DHRS3	GO:0001523(retinoid metabolic process);GO:0003151(outflow tract morphogenesis);GO:0004745(retinol dehydrogenase activity);GO:0005811(lipid droplet);GO:0016020(membrane);GO:0016021(integral component of membrane);GO:0030278(regulation of ossification);GO:0042572(retinol metabolic process);GO:0048385(regulation of retinoic acid receptor signaling pathway);GO:0048387(negative regulation of retinoic acid receptor signaling pathway);GO:0055114(oxidation-reduction process);GO:0060021(roof of mouth development);GO:0060349(bone morphogenesis);GO:0060411(cardiac septum morphogenesis)	00830(Retinol metabolism)	MSTRG.21758

EDN2	GO:0001516(prostaglandin biosynthetic process);GO:0001659(temperature homeostasis);GO:0002690(positive regulation of leukocyte chemotaxis);GO:0003100(regulation of systemic arterial blood pressure by endothelin);GO:0005179(hormone activity);GO:0005576(extracellular region);GO:0005615(extracellular space);GO:0005623(cell);GO:0007204(positive regulation of cytosolic calcium ion concentration);GO:0008284(positive regulation of cell population proliferation);GO:0010460(positive regulation of heart rate);GO:0010469(regulation of signaling receptor activity);GO:0014824(artery smooth muscle contraction);GO:0014826(vein smooth muscle contraction);GO:0019221(cytokine-mediated signaling pathway);GO:0019229(regulation of vasoconstriction);GO:0019722(calcium-mediated signaling);GO:0030593(neutrophil chemotaxis);GO:0031708(endothelin B receptor binding);GO:0042116(macrophage activation);GO:0042310(vasoconstriction);GO:0045987(positive regulation of smooth muscle contraction);GO:0048016(inositol phosphate-mediated signaling);GO:0048246(macrophage chemotaxis);GO:0048286(lung alveolus development);GO:0060585(positive regulation of prostaglandin-endoperoxide synthase activity);GO:0097009(energy homeostasis)	04024(cAMP signaling pathway);04080(Neuroactive ligand-receptor interaction);04270(Vascular smooth muscle contraction);04924(Renin secretion)	MSTRG.22932
SH3D19	GO:0005515(protein binding);GO:0005654(nucleoplasm);GO:0005829(cytosol);GO:0005886(plasma membrane);GO:0007010(cytoskeleton organization);GO:0022604(regulation of cell morphogenesis);GO:0051044(positive regulation of membrane protein ectodomain proteolysis)	NA	MSTRG.25469
PRSS12	GO:0004252(serine-type endopeptidase activity);GO:0005044(scavenger receptor activity);GO:0005886(plasma membrane);GO:0006508(proteolysis);GO:0006887(exocytosis);GO:0006898(receptor-mediated endocytosis);GO:0008233(peptidase activity);GO:0008236(serine-type peptidase activity);GO:0016020(membrane);GO:0016787(hydrolase activity);GO:0030424(axon);GO:0030425(dendrite);GO:0031410(cytoplasmic vesicle);GO:0031638(zymogen activation);GO:0043083(synaptic cleft);GO:0043195(terminal bouton);GO:0045202(synapse)	NA	MSTRG.25658

PLN	GO:0002026(regulation of the force of heart contraction);GO:0005246(calcium channel regulator activity);GO:0005739(mitochondrion);GO:0005783(endoplasmic reticulum);GO:0005789(endoplasmic reticulum membrane);GO:0006816(calcium ion transport);GO:0006874(cellular calcium ion homeostasis);GO:0007219(Notch signaling pathway);GO:0008016(regulation of heart contraction);GO:0010459(negative regulation of heart rate);GO:0010880(regulation of release of sequestered calcium ion into cytosol by sarcoplasmic reticulum);GO:0010881(regulation of cardiac muscle contraction by regulation of the release of sequestered calcium ion);GO:0016020(membrane);GO:0016021(integral component of membrane);GO:0016529(sarcoplasmic reticulum);GO:0031966(mitochondrial membrane);GO:0032780(negative regulation of ATPase activity);GO:0033017(sarcoplasmic reticulum membrane);GO:0042030(ATPase inhibitor activity);GO:0045822(negative regulation of heart contraction);GO:0048738(cardiac muscle tissue development);GO:0051924(regulation of calcium ion transport);GO:0051926(negative regulation of calcium ion transport);GO:0060314(regulation of ryanodine-sensitive calcium-release channel activity);GO:0086004(regulation of cardiac muscle cell contraction);GO:0086023(adenylate cyclase-activating adrenergic receptor signaling pathway involved in heart process);GO:0086092(regulation of the force of heart contraction by cardiac conduction);GO:0090279(regulation of calcium ion import);GO:0090534(calcium ion-transporting ATPase complex);GO:1901020(negative regulation of calcium ion transmembrane transporter activity);GO:1901077(regulation of relaxation of muscle);GO:1901877(negative regulation of calcium ion binding);GO:1901894(regulation of calcium-transporting ATPase activity);GO:1901895(negative regulation of calcium-transporting ATPase activity)	04020(Calcium signaling pathway);04022(cGMP-PKG signaling pathway);04024(cAMP signaling pathway);04261(Adrenergic signaling in cardiomyocytes);04919(Thyroid hormone signaling pathway);05414(Dilated cardiomyopathy (DCM))	MSTRG.407
ENSSSCG00000011290	GO:0016020(membrane);GO:0016021(integral component of membrane)	NA	MSTRG.5679
JAGN1	GO:0002446(neutrophil mediated immunity);GO:0005783(endoplasmic reticulum);GO:0005789(endoplasmic reticulum membrane);GO:0007029(endoplasmic reticulum organization);GO:0016192(vesicle-mediated transport);GO:0030223(neutrophil differentiation);GO:0038158(granulocyte colony-stimulating factor signaling pathway);GO:0050832(defense response to fungus);GO:1990266(neutrophil migration)	04060(Cytokine-cytokine receptor interaction);04657(IL-17 signaling pathway)	MSTRG.6138

CYP2E1	GO:0004497(monooxygenase activity);GO:0005506(iron ion binding);GO:0005737(cytoplasm);GO:0005783(endoplasmic reticulum);GO:0006082(organic acid metabolic process);GO:0006805(xenobiotic metabolic process);GO:0008202(steroid metabolic process);GO:0008392(arachidonic acid epoxygenase activity);GO:0008395(steroid hydroxylase activity);GO:0009617(response to bacterium);GO:0016020(membrane);GO:0016021(integral component of membrane);GO:0016098(monoterpenoid metabolic process);GO:0016491(oxidoreductase activity);GO:0016705(oxidoreductase activity, acting on paired donors, with incorporation or reduction of molecular oxygen);GO:0016712(oxidoreductase activity, acting on paired donors, with incorporation or reduction of molecular oxygen, reduced flavin or flavoprotein as one donor, and incorporation of one atom of oxygen);GO:0017144(drug metabolic process);GO:0019373(epoxygenase P450 pathway);GO:0019899(enzyme binding);GO:0020037(heme binding);GO:0042738(exogenous drug catabolic process);GO:0043231(intracellular membrane-bounded organelle);GO:0046483(heterocycle metabolic process);GO:0046872(metal ion binding);GO:0055114(oxidation-reduction process)	00140(Steroid hormone biosynthesis);00590(Arachidonic acid metabolism);00591(Linoleic acid metabolism);00980(Metabolism of xenobiotics by cytochrome P450);00982(Drug metabolism - cytochrome P450);00983(Drug metabolism - other enzymes);04932(Non-alcoholic fatty liver disease (NAFLD));05204(Chemical carcinogenesis)	MSTRG.9318
